# Clinical, biochemical and molecular characterization of a new case with *FDX2* ‐related mitochondrial disorder: Potential biomarkers and treatment options

**DOI:** 10.1002/jmd2.12408

**Published:** 2024-01-23

**Authors:** Parith Wongkittichote, Cassandra Pantano, Miao He, Xinying Hong, Matthew M. Demczko

**Affiliations:** ^1^ Division of Human Genetics Children's Hospital of Philadelphia Philadelphia Pennsylvania USA; ^2^ Department of Pathology and Laboratory Medicine Children's Hospital of Philadelphia Philadelphia Pennsylvania USA; ^3^ Department of Pediatrics, Faculty of Medicine Ramathibodi Hospital Mahidol University Bangkok Thailand; ^4^ University of Pennsylvania, Perelman School of Medicine Philadelphia Pennsylvania USA

**Keywords:** ferredoxin‐2, iron‐sulfur clusters, mitochondrial disorders, mitochondrial myopathy, episodic, with optic atrophy and reversible leukoencephalopathy, urine organic acid analysis

## Abstract

Ferredoxin‐2 (FDX2) is an electron transport protein required for iron–sulfur clusters biosynthesis. Pathogenic variants in *FDX2* have been associated with autosomal recessive *FDX2*‐related disorder characterized by mitochondrial myopathy with or without optic atrophy and leukoencephalopathy. We described a new case harboring compound heterozygous variants in *FDX2* who presented with recurrent rhabdomyolysis with severe episodes affecting respiratory muscle. Biochemical analysis of the patients revealed hyperexcretion of 2‐hydroxyadipic acid, along with previously reported biochemical abnormalities. The proband demonstrated increased lactate and creatine kinase (CK) with increased amount of glucose infusion. Lactate and CK drastically decreased when parenteral nutrition containing high protein and lipid contents with low glucose was initiated. Overall, we described a new case of *FDX2*‐related disorder and compare clinical, biochemical and molecular findings with previously reported cases. We demonstrated that 2‐hydroxyadipic acid biomarker could be used as an adjunct biomarker for *FDX2*‐related disorder and the use of parenteral nutrition as a treatment option for the patient with *FDX2*‐related disorder during rhabdomyolysis episode.

**Highlights:**

2‐Hydroxyadipic acid can serve as a potential adjunct biomarker for iron‐sulfur assembly defects and lipoic acid biosynthesis disorders. Parenteral nutrition containing high lipid and protein content could be used to reverse acute rhabdomyolysis episodes in the patients with FDX2‐related disorder.

## 1 INTRODUCTION

**FIGURE 1:**
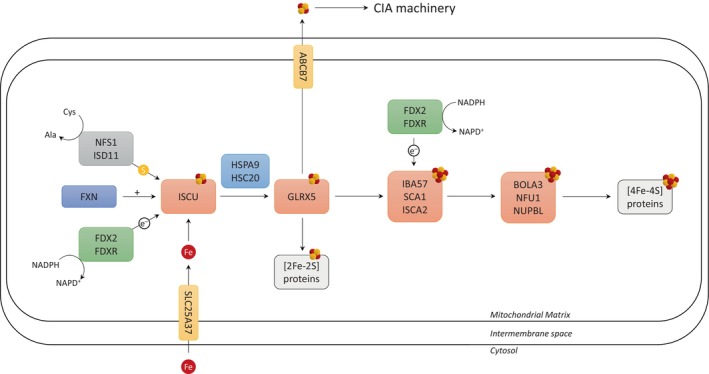
Schematic representation of Fe‐S cluster assembly. Adapted from.^3^, ^4^

Iron‐sulfur clusters (Fe‐S) are one of the most ubiquitous cofactors for multiple enzymes with various biological function.^1^, ^2^ Mitochondrial biosynthesis of Fe‐S is a complex process with tight regulations.^3^, ^4^ Sulfur atoms donated by cysteine via the reaction of cysteine desulfurase complex, iron atoms and electrons combine to form [2Fe‐2S] clusters using multiple scaffolding and transport proteins (Figure 1). [2Fe‐2S] clusters are transported to target proteins or further converted to [4Fe‐4S] clusters.

Although very rare, mitochondrial Fe‐S biosynthesis defects are an emerging group of metabolic disorders with various phenotype and broad clinical spectrum.^5^ Among those, the defect in electron transport protein, ferredoxin‐2 (FDX2), encoded by *FDX2* (formerly known as *FDX1L*), causes autosomal recessive episodic mitochondrial myopathy with or without optic atrophy and reversible leukoencephalopathy (MEOAL, MIM# 251900).^6^ To date, 10 patients from 6 families have been reported with *FDX2*‐related disorder.^6^, ^7^, ^8^, ^9^, ^10^, ^11^ Clinical phenotype included progressive myopathy, rhabdomyolysis with variable onset and severity, peripheral neuropathy, and reversible leukoencephalopathy. Optic atrophy, neurodevelopmental abnormalities, microcytic anemia, neutropenia, hypothyroidism have been reported in some patients. Biochemical abnormalities include lactic acidosis, and hyperexcretion of lactate, ketones, tricarboxylic acid (TCA) cycle metabolites, and 3‐methylglutaconic acid in urine organic acid analysis.^6^, ^7^, ^10^, ^11^ Mitochondrial respiratory chain analysis revealed decreased activities of several mitochondrial complexes and mitochondrial aconitase.^6^

Here, we report a patient with *FDX2*‐related disorder who presented with severe rhabdomyolysis affecting respiratory muscle. Urine organic acid analysis revealed increased 2‐hydroxyadipic acid (2‐HAA), a potential biomarker for MEOAL and other Fe‐S biosynthesis defects. Despite the severe presentation, the patient recovers quickly with parental nutrition containing high protein content, which is a viable treatment option during acute metabolic crisis.

### 1.1 Case description

The proband is a 10‐year‐old male with Eastern European ancestry. He was born full term with uncomplicated pregnancy and delivery. He had normal developmental milestones but was noticed to have decreased exercise tolerance compared to other children.

At the age of 6 years, he developed dyspnea and weakness after exercise. His examination at that time was notable for positive Gower's sign and hyperlordosis. He had multiple episodes of rhabdomyolysis presenting with lower extremity weakness and elevated creatine kinase (CK) following initial evaluation. At 9 years of age, he developed severe rhabdomyolysis episode leading to prolonged respiratory failure requiring tracheostomy. Brain magnetic resonance imaging (MRI) was normal. After 2 months of initial recovery, he was subsequently transferred to our facility for further investigation. Initial biochemical analysis during recovery state revealed normal CK and plasma amino acids, including normal glycine, and slightly increased plasma acetylcarnitine (C2) at 24.78 μmol/L (RR 4.21–20.60 μmol/L). Plasma growth differentiation factor 15 (GDF15) was significantly elevated at 5914 pg/mL (reference range (RR) 0–750 pg/mL). Clinical exome sequencing (Invitae, San Francisco, California) revealed compound heterozygous variants in *FDX2* (NM_001397406.1), designated as c.1A > T (p.Met1?) and c.146‐2A > G. The variant p.Met1? has been reported previously in the patients with *FDX2*‐related disorder.^6^, ^8^ The variant c.146‐2A > G is predicted to cause abnormal splicing, disrupting protein function. Both variants have been reported at very low frequency in general population, supporting their pathogenicity. He was subsequently decannulated and discharged with home regimen of sodium bicarbonate and mitochondrial cocktail comprising of n‐acetylcysteine, niacin, biotin and coenzyme Q10.

**FIGURE 2:**
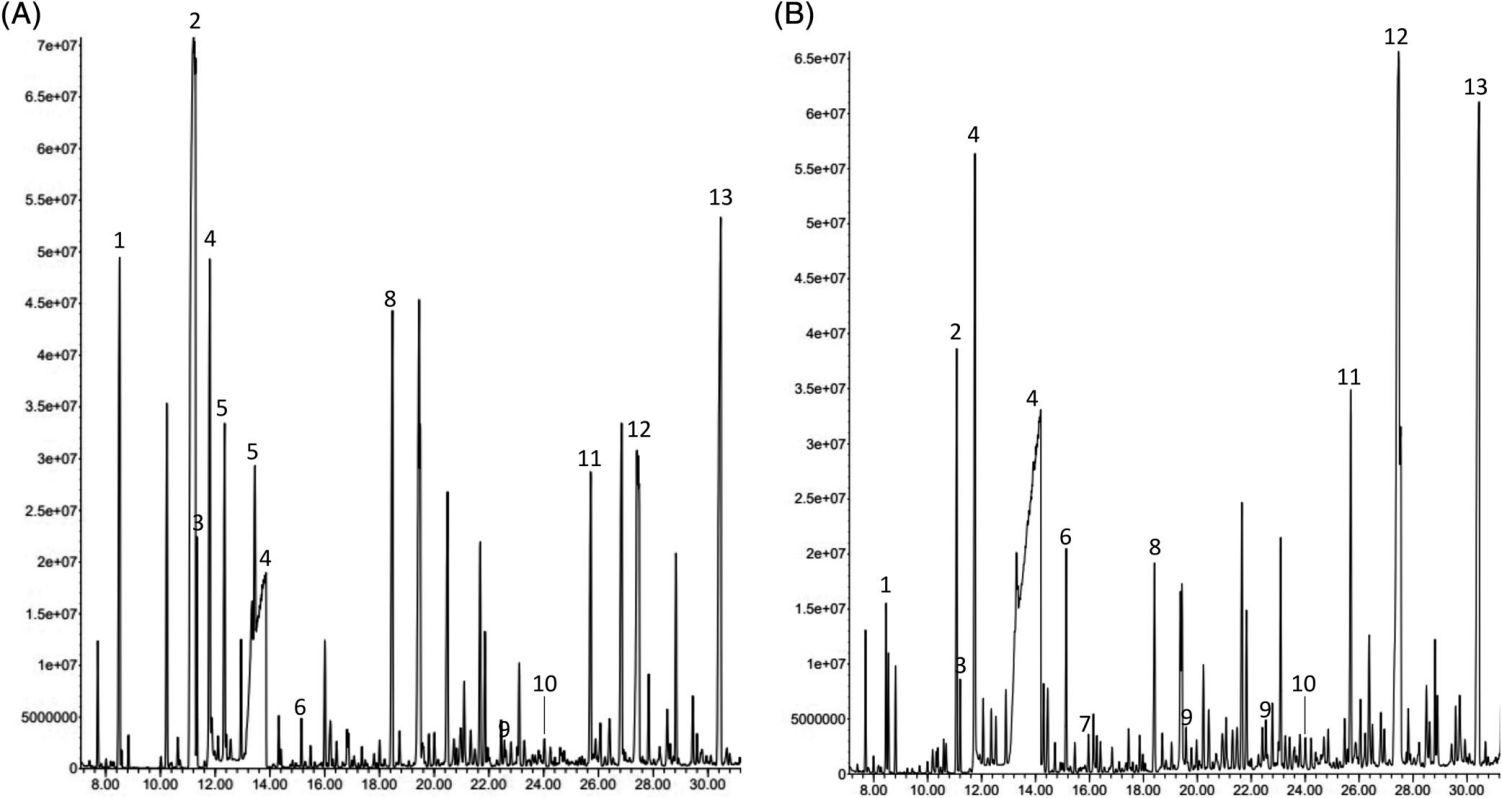
Urine organic acid profile of the proband. Total iron chromatograms of the proband during decompensation (A) and recovery phase (B). Numbers indicate the following metabolites: (1) lactate, (2) 3‐hydroxybutyrate, (3) 2‐hydroxyisovaleric acid, (4) urea, (5) acetoacetate, (6) succinic acid, (7) fumaric acid, (8) 3‐methylglutaconic acid, (9) 2‐ketoglutaric acid, (10) 2‐hydroxyadipic acid, (11) aconitic acid, (12) citric acid, (13) undecanedioic acid (internal standard). Acquired on Agilent Technologies Gas Chromatography Mass Spectrometer 5977B (Column: HP‐5MS). Data were analyzed by Agilent MassHunter Workstation version 10.0.

At the age of 10 years, he developed another episode involving respiratory muscle after receiving tetanus vaccine. He was noted to have lactic acidosis (11.9 mmol/L, RR 0.5–2.2 mmol/L), ketosis (plasma 3‐hydroxybutyrate 0.9 mmol/L, RR 0.1–0.3 mmol/L) and elevated CK (21 005 U/L, RR 55–215 U/L). Intravenous sodium bicarbonate, and intravenous fluid containing 5% dextrose were initiated with subsequent improvement of acidosis. Metabolic evaluation during decompensation demonstrated, again, normal plasma amino acids and slightly increased plasma C2. Urine organic acid analysis revealed hyperexcretion of lactate, ketones, TCA cycle metabolites, 3‐methylglutaconic acid (94.1 mmol/mol creatinine, RR 0–15 mmol/mol creatinine), 2‐hydroxyisovaleric acid (2‐HIA, 18.1 mmol/mol creatinine) and 2‐HAA (4.1 mmol/mol creatinine, RR <1 mmol/mol creatinine) (Figure 2A).

**FIGURE 3:**
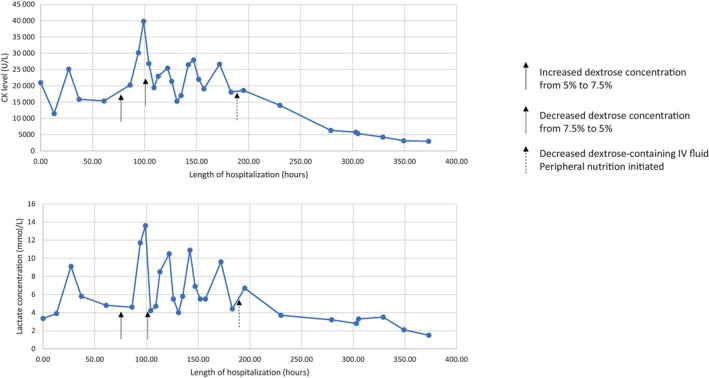
Treatment response to parental nutrition containing high‐protein and lipid contents.

Despite continuous intravenous dextrose fluid, his CK continued to be fluctuated between 15 358 and 39 814 U/L. Increased dextrose concentration to 7.5% led to subsequent increase of lactate level. Parenteral nutrition with protein and lipid with 5% dextrose concentration were then initiated. He responded dramatically with decreased CK and lactate levels (Figure 3). Upon follow‐up visit 2 weeks after discharged, he achieved resolution of the symptoms and normalization of CK and lactate. Urine organic acid analysis obtained at the follow‐up visit revealed persistently increased 3‐methylglutaconic acid (40.3 mmol/mol creatinine), 2‐HIA (6.3 mmol/mol creatinine), 2‐HAA (3.5 mmol/mol creatinine) (Figure 2B).

## 2 DISCUSSION

We describe a proband with *FDX2*‐related disorder who presented with profound rhabdomyolysis affecting respiratory function. To date, 11 patients with *FDX2*‐related disorder from 7 families have been reported, including the proband (Table 1).^6^, ^7^, ^8^, ^9^, ^10^ Most of the patients presented with progressive myopathy (11/11) and acute rhabdomyolysis (6/11). Extra‐muscular manifestations including optic atrophy and hematologic complications have been reported in two Brazilian families harboring homozygous c.422C > T (p.Pro141Leu) but have not been reported elsewhere.^9^ The previously reported patients with predominant muscular presentation harbor the variants affecting the initiator codon.^6^, ^7^, ^8^, ^10^, ^11^ The proband is the first reported patient with compound heterozygous variants. The patients harboring missense variants affecting initiator codon did not demonstrate extramuscular involvement. It is possible that pathogenic variants in *FDX2* may cause two distinct disease entities: a milder phenotype presenting with episodic rhabdomyolysis and progressive myopathy, and a more severe phenotype with multisystemic involvement, described as MEOAL. The former phenotype has been associated with the patients who harbor initiator codon variant in at least one allele, while other pathogenic variants which severely impact protein function may be associated with MEOAL phenotype. This phenomenon has been observed in *COQ7*‐related disorder.^12^, ^13^ Larger cohort of patients with *FDX2*‐related disorder are needed to establish genotype–phenotype correlation.

**TABLE 1:** Clinical, biochemical and molecular characterization of the reported cases of MEOAL.

	Spiegel et al. (2014)^6^	Lebigot et al. (2017)^8^ and Montealegre et al. (2022)^7^	Gurgel‐Giannetti et al. (2018)^9^	Aggarwal et al. (2021)^10^	Gkiourtzis et al. (2023)^11^	Current report
Age at initial presentation	12 years	5 months	<6 months–1.3 years	10 years	13.5 years	6 years
Number of individuals	1	1	6	1	1	1
Sex	Female	Female	3 Males 3 Females	Male	Female	Male
Genotype	c.1A > T (p.Met1?)	c.1A > T (p.Met4?)	c.422C > T (p.Pro141Leu)	c.3G > T (p.Met1?)	c.3G > T (p.Met1?)	c.1A > T (p.Met1?); c.146‐2A > G
Zygosity	Homozygous	Homozygous	Homozygous	Homozygous	Homozygous	Compound heterozygous
Progressive myopathy	+	+	6/6	+	+	+
Acute Rhabdomyolysis	+	+	1/6	+	+	+
Abnormal brain MRI	ND	−	4/6	−	−	−
Optic atrophy	ND	ND	6/6	−	−	−
Diabetes mellitus	ND	ND	2/6	ND	ND	−
Subclinical hypothyroidism	ND	ND	3/5	ND	ND	−
Microcytic anemia	ND	ND	4/6	−	−	−
Neutropenia	ND	ND	4/6	−	−	−
Plasma lactate (mmol/L)	7.8	23	ND	20	21	11.9
Peak CK (U/L)	31 000	105 000	3000	>1 000 000	319 990	39 814
Urine organic acid	Increased 3‐methylglutaconic acid	Increased lactate and 2‐ketoglutarate	ND	Increased lactate, pyruvate, and ketones	Increased lactate, ketones, TCA cycle, BCAA and tyrosine metabolites, 3‐methylglutaconic acid	Increased lactate, ketones, TCA cycle metabolites, 3‐methylglutaconic acid, and 2‐hydroxyadipic acid
Plasma amino acid	ND	Constant elevation of alanine and mild decrease of BCAAs	ND	Intermittent increase of isoleucine, valine, leucine and allo‐isoleucine	Increased methionine	Normal
Treatment	CoQ10, thiamine and riboflavin	ND	ND	CoQ10, lipoic acid, leucovorin, levocarnitine, MCT oil and B‐complex vitamins	coQ10, biotin, thiamine, and riboflavin	n‐acetylcysteine, niacin, biotin and CoQ10

Multiple dehydrogenase complexes, including 2‐ketoglutarate dehydrogenase (KGDH), pyruvate dehydrogenase (PDH), branched‐chain keto‐acid dehydrogenase (BCKDH), and 2‐ketoadipate dehydrogenase (OADH), are lipoic‐acid dependent enzymes composed of three subunits: E1, E2 and E3.^14^, ^15^, ^16^ Lipoic acid in E2 subunit is used for dehydrogenation reaction performed by E1 subunit, while E3 subunit regenerates oxidized lipoic acid.^15^ In human, lipoic acid biosynthesis requires multiple enzymes including lipoyltransferase 1 and 2 (LIPT1, LIPT2) and lipoic acid synthase (LIAS).^17^, ^18^ LIAS requires [4Fe‐4S] for its appropriate function; therefore, the defect in [4Fe‐4S] cluster formation disrupts lipoic acid biosynthesis and subsequently the activities of lipoic‐acid dependent dehydrogenase complexes.^17^, ^18^

2‐Ketoadipic acid (2KAA) is a metabolite in lysine and tryptophan catabolism.^19^ 2KAA is catalyzed to glutaryl‐CoA by the reaction of 2‐oxoadipate dehydrogenase (OADH), a lesser‐known dehydrogenase complex. Its E1 subunit, encoded by *DBTKD1*, is homologous to E1 subunit of KGDH.^19^ OADH shares its E2 subunit with KGDH and E3 with other dehydrogenase complexes.^19^, ^20^, ^21^ Biallelic pathogenic variants in *DBTKD1* have been associated with autosomal recessive alpha‐aminoadipic and alpha‐ketoadipic aciduria (AAKAD).^22^ Although clinical significance of AAKAD is unclear, the patients with AAKAD exhibited hyperexcretion of 2‐aminoadipic acid, 2‐KAA and 2‐HAA.^23^ It is also unclear whether the formation of 2‐HAA is from the enzymatic reaction similar to the conversion of 2‐ketoglutaric to 2‐hydroxyglutaric acid,^24^ or occurs during analytic process.

Interestingly, previous studies showed that lipoylation of PDH and KGDH in the fibroblasts and myoblasts of the patients with MEOAL are normal.^7^, ^8^ However, recent study showed that reduced FDX2 expression led to the decrease in Fe‐S biosynthesis and subsequent abnormal lipoylation.^25^ Untargeted metabolomic analysis of the blood specimen from the patient with MEOAL revealed branched‐chain keto‐acids (2‐HIA, 2‐hydroxy‐3‐methylvaleric acids), and TCA cycle intermediates, which indicate the disruption of BCKDH and KGDH activities.^10^ 2‐HAA has been reported in urine organic acid analysis of the patients with DLD deficiency and Fe‐S assembly defects due to *IBA57* and *NFU1*.^26^, ^27^, ^28^ 2‐HAA has been speculated to be potential biomarker for DLD deficiency, lipoic acid biosynthesis disorders, and Fe‐S assembly defects.^17^, ^27^, ^29^ We demonstrate that 2‐HAA, when presents concurrently with branched‐chain keto‐acids, should raise the concern for multiple dehydrogenase complexes deficiency caused by DLD deficiency, lipoic acid biosynthesis disorders, and Fe‐S assembly defects. In the proband, the levels of 2‐HAA are not largely different during decompensated and well periods, which represents its role as a diagnostic marker. The levels of 2‐HAA does not seem to correlate with the disease activity, however more studies are needed to establish its potentials as disease monitoring tool.

Previous study showed that patient with *FDX2*‐related disorder is sensitive to high dextrose fluid, leading to increased lactate production.^10^, ^11^ The proband also demonstrated increased blood lactate with the increase of glucose infusion rate. This phenomenon has been known in PDH deficiency, and in fact, the patients with PDH deficiency responded to ketogenic diet.^30^, ^31^ Intralipid has been shown to improve acute rhabdomyolysis episodes.^10^ We demonstrated the use of parenteral nutrition containing high lipid and protein content to reverse acute rhabdomyolysis and lactic acidosis. Despite the possibility of BCKDH dysfunction, the proband improved clinically and biochemically without abnormalities in plasma amino acids.

Overall, we describe a novel case of *FDX2*‐related disorder who presented with rhabdomyolysis episode and subsequent respiratory failure who responded to high fat and protein parenteral nutrition. We also demonstrated the use of 2‐HAA as an adjunct marker for *FDX2*‐related disorder and, possibly, other Fe‐S assembly defects and lipoic acid biosynthesis disorders, especially when it presents with BCAA metabolites. We also demonstrated certain degree of genotype–phenotype correlation, although larger cohort of patients are needed to establish genotype–phenotype correlation and the performance of diagnostic markers.

## AUTHOR CONTRIBUTIONS

PW and MMD designed and conceptualized the study. CP and MMD performed clinical analysis of the patient. PW, XH and MH performed biochemical analysis of the patient. PW, CP and MMD performed variant analysis. PW drafted the manuscript. All authors were involved with revising the manuscript. CP and MMD obtained consent. PW and MMD supervised the study.

## FUNDING INFORMATION

Not applicable.

## CONFLICT OF INTEREST STATEMENT

Parith Wongkittichote, Cassandra Pantano, Miao He, Xinying Hong, Matthew M. Demczko declare that they have no conflict of interest.

## ETHICS STATEMENT

No interventions performed on patients and no additional biological specimens collected from participants.

## PATIENT CONSENT STATEMENT

All procedures followed were in accordance with the ethical standards of the responsible committee on human experimentation (institutional and national) and with the Helsinki Declaration of 1975, as revised in 2000 (5). Informed consent was obtained from all patients for being included in the study.

## Data Availability

Not applicable.
